# Myocardial expression of somatotropic axis, adrenergic signalling, and calcium handling genes in heart failure with preserved ejection fraction and heart failure with reduced ejection fraction

**DOI:** 10.1002/ehf2.13067

**Published:** 2021-01-29

**Authors:** Roberta D'Assante, Michele Arcopinto, Giuseppe Rengo, Andrea Salzano, Marion Walser, Giuseppina Gambino, Maria Gaia Monti, Leonardo Bencivenga, Alberto M. Marra, David N. Åberg, Carlo De Vincentiis, Andrea Ballotta, Eduardo Bossone, Jörgen Isgaard, Antonio Cittadini

**Affiliations:** ^1^ Department of Translational Medical Sciences Federico II University of Naples Via Pansini 5 Naples 80138 Italy; ^2^ Istituti Clinici Scientifici Maugeri SpA Società Benefit (ICS Maugeri SpA SB) – IRCCS ‑ Scientific Institute of Telese Terme Telese Terme Italy; ^3^ IRCCS SDN, Diagnostic and Nuclear Research Institute Naples Italy; ^4^ Department of Internal Medicine Sahlgrenska Academy at the University of Gothenburg Gothenburg Sweden; ^5^ Department of Cardiothoracic and Vascular Anesthesia and Intensive Care IRCCS Policlinico San Donato Milan Italy; ^6^ Division of Cardiology A. Cardarelli Hospital Naples Italy

**Keywords:** Heart failure, Growth hormone, Insulin‐like growth factor 1, HFpEF, Adrenergic signalling, Calcium handling

## Abstract

**Aims:**

Limited data are available regarding cardiac expression of molecules involved in heart failure (HF) pathophysiology. The majority of the studies have focused on end‐stage HF with reduced ejection fraction (HFrEF) without comparison with healthy subjects, while no data are available with regard to HF with preserved ejection fraction (HFpEF). HFpEF is a condition whose multiple pathophysiological mechanisms are still not fully defined, with many proposed hypotheses remaining speculative due to limited access to human heart tissue. This study aimed at evaluating cardiac expression levels of key genes of interest in human biopsy samples from patients affected with HFrEF and HFpEF in order to possibly point out distinct phenotypes.

**Methods and results:**

Total RNA was extracted from left ventricular cardiac biopsies collected from stable patients with HFrEF (*n* = 6) and HFpEF (*n* = 7) and healthy subjects (*n* = 9) undergoing elective cardiac surgery for valvular replacement, mitral valvuloplasty, aortic surgery, or coronary artery bypass. Real‐time PCR was performed to evaluate the mRNA expression levels of genes involved in somatotropic axis regulation [IGF‐1, IGF‐1 receptor (IGF‐1R), and GH receptor (GHR)], in adrenergic signalling (GRK2, GRK5, ADRB1, and ADRB2), in myocardial calcium handling (SERCA2), and in TNF‐α. Patients with HFrEF and HFpEF showed reduced serum IGF‐1 circulating levels when compared with controls (102 ± 35.6, 138 ± 11.5, and 160 ± 13.2 ng/mL, *P* < 0.001, respectively). At myocardial level, HFrEF showed significant decreased GHR and increased IGF‐1R expressions when compared with HFpEF and controls (0.54 ± 0.27, 0.94 ± 0.25, and 0.84 ± 0.2, *P* < 0.05 and 1.52 ± 0.9, 1.06 ± 0.21, and 0.72 ± 0.12, *P* < 0.05, respectively), while no differences in the local expression of IGF‐1 mRNA were detected among the groups (0.80 ± 0.45, 0.97 ± 0.18, and 0.63 ± 0.23, *P* = 0.09, respectively). With regard to calcium handling and adrenergic signalling, HFrEF displayed significant decreased levels of SERCA2 (0.19 ± 0.39, 0.82 ± 0.15, and 0.87 ± 0.32, *P* < 0.01) and increased levels of GRK2 (3.45 ± 2.94, 0.93 ± 0.12, and 0.80 ± 0.14, *P* < 0.01) and GRK5 (1.32 ± 0.70, 0.71 ± 0.14, and 0.77 ± 0.15, *P* < 0.05), while no significant difference was found in ADRB1 (0.66 ± 0.4, 0.83 ± 0.3, and 0.86 ± 0.4) and ADRB2 mRNA expression (0.65 ± 0.3, 0.66 ± 0.2, and 0.68 ± 0.1) when compared with HFpEF and controls. Finally, no changes in the local expression of TNF‐α were detected among groups.

**Conclusions:**

Heart failure with reduced ejection fraction and HFpEF patients with stable clinical condition display a distinct molecular milieu of genes involved in somatotropic axis regulation, calcium handling, and adrenergic derangement at a myocardial level. The unique opportunity to compare these results with a control group, as reference population, may contribute to better understand HF pathophysiology and to identify novel potential therapeutic targets that could be modulated to improve ventricular function in patients with HF.

## Introduction

In the last decades, a relevant piece of scientific literature has contributed to increase the knowledge of the molecular mechanisms underlying heart failure (HF) onset and progression, which enabled the identification of novel therapeutic targets. Mounting evidence supports the role of anabolic deficiencies in HF pathophysiology,^1^ a key player being the impairment of the growth hormone/insulin‐like growth factor 1 (GH/IGF‐1) axis,^2^, ^3^ which molecular regulation still represents a huge gap in evidence. However, the vast majority of investigations reporting data on circulating levels of GH, IGF‐1, or binding proteins have been conducted in HF with reduced ejection fraction (HFrEF), while limited evidence is available in the setting of HF with preserved ejection fraction (HFpEF). HF progression is also known to be influenced by various factors including the expression of proteins related to hyperactive adrenergic signalling and impaired myocardial contractility, which pharmacological modulation is part of HF therapeutic armamentarium. Increased G protein‐coupled receptor kinases (GRKs) levels/activity and reduced sarco–endoplasmic reticulum calcium ATPase (SERCA2) expression, widely implicated in HF‐dependent beta‐adrenergic receptor (ADRB) dysfunction and altered calcium homoeostasis, play a pivotal role in HFrEF pathophysiology, but their involvement in HFpEF remains undefined.^4^ HFpEF appears a disease with multiple proposed pathophysiological mechanisms, though many remain speculative due to limited access to human heart tissue. Further, an additional limitation is that cardiac biopsies are commonly collected from end‐stage HF patients, often treated with inotropic agents or placed on heart transplant waiting lists, and frequently without appropriate healthy controls.

## Methods

Thirteen HF patients undergoing elective cardiac surgery for valvular replacement, mitral valvuloplasty, aortic surgery, or coronary artery bypass, assigned to HFrEF (left ventricular ejection fraction ≤ 40%; *n* = 6) and HFpEF (left ventricular ejection fraction ≥ 50%; *n* = 7) groups according to 2016 European Society of Cardiology Guidelines,^5^ were enrolled at the Department of Cardiac Surgery, IRCCS Policlinico San Donato between 2014 and 2017. All HFrEF patients were suffering from ischaemic heart disease compared with only one in the HFpEF group, in line with the well‐known different aetiologies of HFpEF and HFrEF, with ischaemic aetiology more prevalent in HFrEF than HFpEF. Moreover, all HFpEF patients presented diabetes (*n* = 3) and hypertension (*n* = 6) as major co‐morbidities. For the purpose of the study, patients with ejection fraction between 40% and 50% were intentionally excluded. Nine subjects with normal cardiac structure and function, without symptoms or sign of HF, undergoing thoracic surgery were recruited as healthy controls. At the time of enrolment, all participants underwent a complete clinical examination, including New York Heart Association (NYHA) functional class assessment and transthoracic echocardiography, and peripheral blood samples were collected. Cardiac biopsies were obtained during surgical procedures by highly qualified and expert surgeons following the maximum safety standards. Samples with a minimum size of 200 mm^3^ were collected from the apical portion of the left ventricle or the junctional portion between the free wall of the left ventricle and the interventricular septum (IVS). Each sample was taken by direct vision, through the aortic valve, and with the scalpel. No full‐thickness samples were taken, and no biopsy needle was used. The techniques and the modalities were exactly the same adopted to remove the muscle spur in the context of obstructive hypertrophic cardiomyopathy. No histopathologic evaluation was performed.

Afterwards, each sample was transferred in a sterile tube containing RNAlater (Qiagen, Valencia, CA, USA) and stored at −80°C until assayed. Total RNA was extracted from cardiac biopsies through TRI Reagent kit (Sigma, St. Louis, MO, USA). Real‐time PCR (TaqMan Gene Expression Assays, Applied Biosystems, Foster City, CA, USA) was performed using predesigned TaqMan^®^ Gene Expression Assays to evaluate the mRNA expression levels of genes involved in somatotropic axis regulation [IGF‐1, IGF‐1 receptor (IGF‐1R), and GH receptor (GHR)], adrenergic signalling (GRK2, GRK5, ADRB1, and ADRB2), myocardial calcium handling (SERCA2), and tumour necrosis factor‐alpha (TNF‐α). Each sample was analysed in duplicate. The relative comparative C_T_ method was used to analyse the quantitative PCR data (Sequence Detector User Bulletin #2, Applied Biosystems, Foster City, CA, USA). In the C_T_ method, the amount of target normalized to an endogenous reference and relative to a calibrator sample is given by 2^−ΔΔCT^. The amount of each transcript was normalized to the amount of the reference gene PPIA (cyclophiline A) expressed in the same sample. The calibrator is the same defined stock sample analysed in triplicates in each of the quantitative PCRs. Thus, the values represent arbitrary but linear relative amounts of each transcript.

The distribution of the parameters was tested with the Kolmogorov–Smirnov test. The intergroup differences were tested with the one‐way ANOVA, with Holm correction as appropriate. Correlations between IGF‐1 circulating levels and IGF‐1R mRNA were examined using Spearman rank‐order correlation. A *P* value < 0.05 was considered statistically significant. Statistical analysis was performed using the R statistical programming environment, version 3.5.

The study was approved by the ethics committee (protocol number 2535) and conforms to the ethical guidelines of the 1975 Declaration of Helsinki. All participants gave written informed consent.

## Results

**Figure 1:**
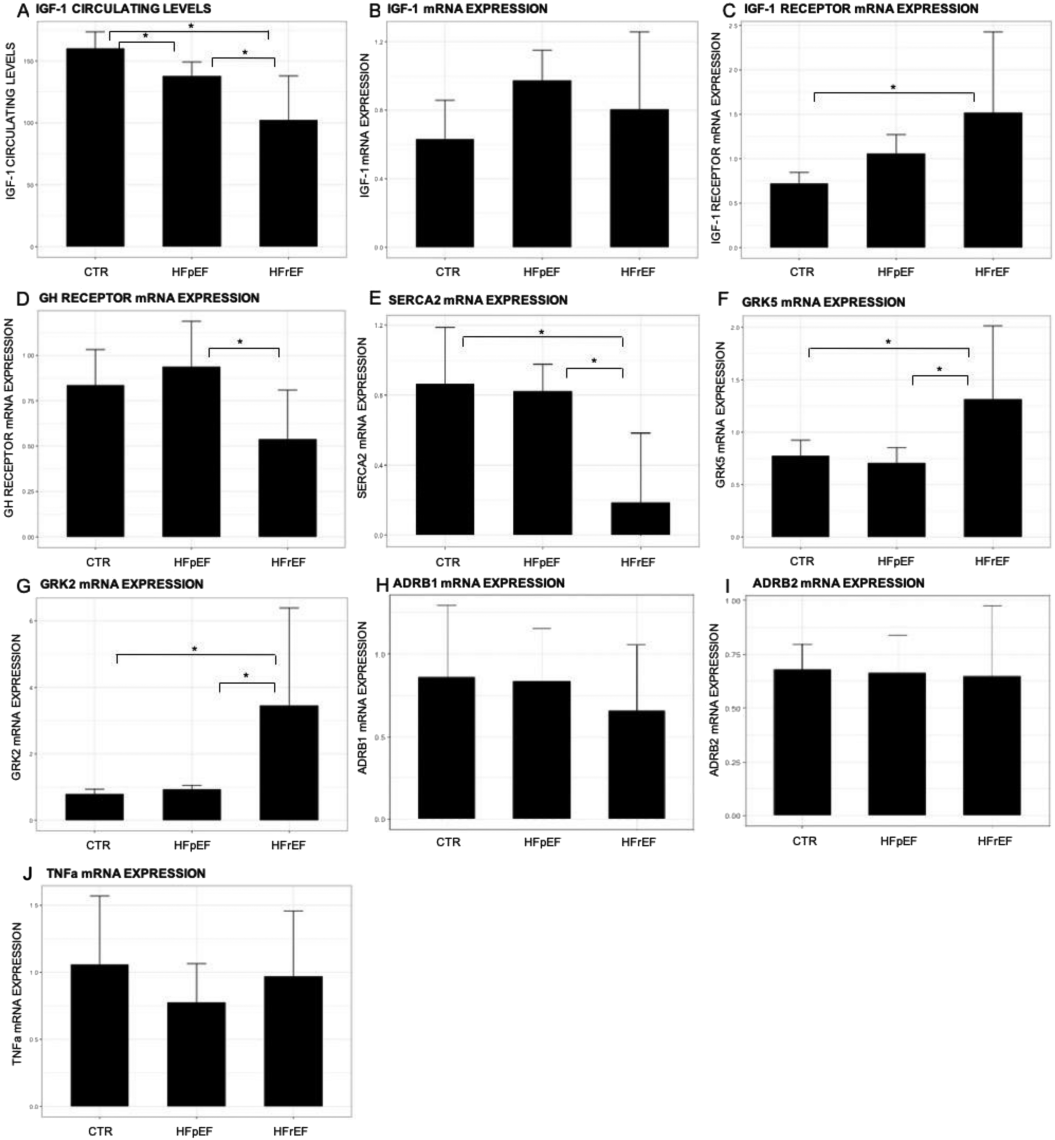
mRNA expression of genes evaluated in cardiac biopsies in controls (CTR), chronic heart failure patients with preserved ejection fraction (HFpEF) or reduced ejection fraction (HFrEF): (A) IGF‐1 circulating levels; (B) IGF‐1 mRNA; (C) IGF‐1R mRNA; (D) GHR mRNA; (E) SERCA2 mRNA; (F) GRK5 mRNA; (G) GRK2 mRNA; (H) ADRB1 mRNA; (I) ADRB2 mRNA; and (J) TNF‐α mRNA. *P < 0.05.

No differences were observed in terms of age, sex, kidney function, and haemoglobin levels among the three study groups. Notably, HFrEF and HFpEF patients displayed comparable stable clinical status, as suggested by the similar NYHA functional class and serum BNP levels, with average values <100 pg/mL (*Table* *1*). Further, both HFpEF and HFrEF patients showed reduced IGF‐1 circulating levels, when compared with controls, with lowest values observed in HFrEF (160 ± 13.2, 138 ± 11.5, and 102 ± 35.6 ng/mL, *P* < 0.001, respectively; *Figure* *1* *A*). Interestingly, no differences in the local expression of IGF‐1 mRNA were detected among the groups (*Figure* *1* *B*). When compared with controls, a significant increased myocardial expression of IGF‐1R was found in HFrEF (0.72 ± 0.12 and 1.52 ± 0.9, *P* < 0.05; *Figure* *1* *C*), while a trend to increase, although not significant, was observed in HFpEF (*Figure* *1* *C*). A negative and significant correlation between IGF‐1 circulating levels and cardiac IGF‐1R expression was found in controls and HFpEF and HFrEF patients (*R* = −0.79, *P* = 0.01; *R* = −0.92, *P* > 0.001; and *R* = −0.85, *P* = 0.03, respectively).

**Table 1:** Clinical characteristics of subjects enrolled in the study

	Values			ANOVA		χ^2^	
Characteristic	CTRL (*n* = 9)	HFpEF (*n* = 7)	HFrEF (*n* = 6)	*F*	*P*	χ^2^	*P*
Age (years)	66.8 ± 14.4	74.3 ± 10.6	64.2 ± 13.3	1.10	0.35		
Sex (M/F)	4/5	5/2	5/1			2.62	0.3
Weight (kg)	75.7 ± 15.9	74.6 ± 12.7	72.2 ± 6.6	0.1	0.9		
Height (m)	1.7 ± 0.1	1.7 ± 0.1	1.7 ± 0.05	0.1	0.9		
BMI (kg/m^2^)	27.1 ± 5.6	26.1 ± 3.7	25.4 ± 2.5	0.2	0.8		
Creatinine (mg/dL)	0.80 ± 0.1	1.1 ± 0.5	1.19 ± 0.8	1.16	0.33		
eGFR (mL/min)	82.2 ± 16.9	76.4 ± 32.8	78.0 ± 33.6	0.09	0.91		
Haemoglobin (g/dL)	13.1 ± 1.3	12.1 ± 1.5	12.8 ± 1.5	0.91	0.42		
BNP (pg/mL)	14.1 ± 5.3* ^,^ **	55.6 ± 27.4	77.5 ± 22.6	20.4	<0.001		
Ejection fraction (%)	60.0 ± 6.6**	61.0 ± 11.4***	32.0 ± 7.8	23.41	<0.001		
NYHA class (I/II/III)	—	2/4/1	1/4/1			0.3	0.9
Time from diagnosis	—	4 ± 2	5 ± 3				0.6
Diastolic dysfunction^a^	5	7	6			7.1	0.03
Ischaemic aetiology	—	1	6			5.5	0.02
Beta‐blockers	4	5	5			1.1	0.5
Type/average dosage of beta‐blockers^b^	Bisoprolol (4)/5.625 mg	Bisoprolol (4)/6.25 mg	Bisoprolol (5)/5.5 mg				
		Carvedilol (1)/12.5 mg b.i.d.	Carvedilol (1)/12.5 mg b.i.d.				
Diuretics	0	1	4			6.1	0.048
ACE/ARBs	3	1	5			4.4	0.1
Calcium channel blockers	0	4	0			10.48	0.01
Antidiabetics	0	3	2			3.88	0.14
MRA	0	0	1			2.79	0.25
Statins	1	2	4			3.3	0.19
OAC/aspirin	1	2	1			0.64	0.72
Type of cardiac surgery	Correction of ascending aorta aneurysm (*n* = 9)	Correction of ascending aorta aneurysm (*n* = 6); CABG (*n* = 1)	Mitral valvuloplasty + CABG (*n* = 2); correction of ascending aorta aneurysm + aortic valve replacement (*n* = 4)				

ACE, angiotensin‐converting enzyme; ARBs, angiotensin receptor blockers; BMI, body mass index; BNP, brain natriuretic peptides; CABG, coronary artery bypass graft; CTRL, non‐heart failure patients; eGFR, estimated glomerular filtration rate; HFpEF, heart failure with preserved ejection fraction; HFrEF, heart failure with reduced ejection fraction; MRA, mineralocorticoid receptor antagonists; NYHA, New York Heart Association; OAC, oral anticoagulant.

^a^ Diastolic dysfunction was established according to the ESC guidelines for the diagnosis and treatment of acute and chronic heart failure 2012: the Task Force for the Diagnosis and Treatment of Acute and Chronic Heart Failure 2012 of the European Society of Cardiology. Developed in collaboration with the Heart Failure Association (HFA) of the ESC (Eur J Heart Fail. 2012 Aug; 14 (8): 803–69. doi: 10.1093/eurjhf/hfs105).

^b^ Beta‐blockers up‐titrated to target dose.

* *P* value comparing CTRL vs. HFpEF.

** *P* value comparing CTRL vs. HFrEF.

*** *P* value comparing HFpEF vs. HFrEF.

With regard to GHR, a significantly reduced expression was observed in HFrEF when compared with HFpEF (0.54 ± 0.27 and 0.94 ± 0.25, *P* < 0.05, *Figure* *1* *D*). Further, HFrEF revealed a marked decrease in cardiac SERCA2 expression compared with controls and HFpEF (0.19 ± 0.39, 0.87 ± 0.32, and 0.82 ± 0.15, *P* < 0.05, respectively; *Figure* *1* *E*). Of note, no differences were observed between controls and HFpEF. With regard to adrenergic signalling, cardiac mRNA levels of GRK5 (1.32 ± 0.70, 0.71 ± 0.14, and 0.77 ± 0.15, *P* < 0.05) and GRK2 (3.45 ± 2.94, 0.93 ± 0.12, and 0.80 ± 0.14, *P* < 0.01) were significantly increased in HFrEF when compared with HFpEF and controls, respectively (*Figure* *1* *F–G*). ADRB1 expression showed a trend to decrease (~24%), although not significant, in HFrEF (0.66 ± 0.4) when compared with controls (0.86 ± 0.4), while ADRB2 expression levels were comparable between HFrEF (0.65 ± 0.3) and controls (0.68 ± 0.1). In HFpEF, the expression levels of ADRB1 (0.83 ± 0.3) and ADRB2 (0.66 ± 0.2) are comparable with that measured in healthy subjects (*Figure* *1* *H–I*).

Finally, no changes in the local expression of TNF‐α were detected among groups (*Figure* *1* *J*).

## Aims

Taking advantage of the availability of left ventricular cardiac biopsies obtained from stable chronic HF patients, the aim of the present study was to compare, for the first time, to the best of our knowledge, cardiac expression levels of genes involved in the somatotropic axis regulation, adrenergic signalling, and calcium handling, among HFrEF and HFpEF patients and a control group, as reference population.

## Conclusions

This is the first investigation dwelling upon the comparison of myocardial expression of the somatotropic axis, adrenergic signalling, and calcium handling genes among HFrEF and HFpEF patients and healthy controls. HFrEF displays significantly increased levels of GRK2 and GRK5, consistent with the well‐known HF‐dependent sympathetic nervous system overdrive, which becomes detrimental in the long term and facilitates HF progression. Cardiomyocyte beta‐adrenergic receptor hyperactivation represents a trigger for GRK up‐regulation, which, in turn, activates the processes of receptor desensitization/down‐regulation, thus resulting in dysfunctional adrenergic signalling.^6^, ^7^, ^8^ Moreover, the results obtained on beta‐adrenergic receptors 1 and 2 expression in HFrEF are consistent with a previous report indicating that in human biopsies from HFrEF patients, ADRB2 levels do not show any HF‐related change, while a decrease in ADRB1 mRNA expression is evident only in patients with advanced NYHA class.^9^ Indeed, our study population consisted of mildly symptomatic HF patients, mostly NYHA Class II, under beta‐blocker therapy that may interfere with beta‐adrenergic receptor expression and signalling.

Of relevance, similar molecular abnormalities were not detected in HFpEF, whose pathophysiology is more related to the presence of co‐morbidities and systemic inflammation, allowing to better understand the results of randomized clinical trials that failed to demonstrate a beneficial effect on survival exerted by beta‐blockers in HFpEF.^10^

While a vast magnitude of studies, conducted in both animal models and in human cardiac tissue of HFrEF patients, indicates that SERCA2a expression/activity is impaired in HF, leading to an altered cardiac calcium homoeostasis and consequent cardiomyocyte reduced contractility, only one study had evaluated expression patterns of genes related to myocardial contractile function in HFpEF patients, selected with different criteria, and considering other genes.^11^ With regard to SERCA2, our findings could contribute to better understand the differences in the contractile capacity between patients with HFrEF and HFpEF. Indeed, HFrEF displays a significant decrease in cardiac SERCA2 levels compared with HFpEF, suggesting that calcium transients might be faster in HFpEF, resulting in an increased calcium reuptake and preserved intracellular calcium sensitivity.

With regard to the GH/IGF‐1 axis, our results indicate that HFrEF and HFpEF exhibit a different pattern of gene expression, while circulating IGF‐1 levels are equally reduced, regardless of ejection fraction. Indeed, when compared with HFpEF and healthy controls, HFrEF showed a significant decreased GHR and an increased IGF‐1R expression. Such divergent behaviour of GHR and IGF‐1 receptor mRNA in HF has been described in other pathological conditions, including diabetes and malnutrition, in which the two mRNAs are not coordinately regulated in all tissues studied.^12^ Moreover, it has been also documented that HFrEF and HFpEF may show a different behaviour regarding the anabolic drive: altered in HFrEF and unmodified in HFpEF.^13^, ^14^ On the other hand, the increased cardiac expression of IGF‐1R in HFrEF may reflect a compensatory mechanism in response to low circulating IGF‐1 levels, in order to augment myocardial hormone uptake and the activation of downstream molecular pathways. These observations, as well as the local increase in IGF‐1 mRNA and the negative correlation between IGF‐1 circulating levels and IGF‐1R mRNA, are in line with our recent evidence of myocardial increased utilization of IGF‐1 in HFrEF.^15^ HFpEF showed a different pattern with a mild reduction of IGF‐1 circulating levels and non‐significant changes in the myocardial expression of IGF‐1, IGF‐1R, and GHR mRNAs when compared with healthy controls. The similar cardiac expression of TNF‐α found across the study groups confirms a mild‐to‐moderate HF status where the myocardial catabolic drive has not yet taken over the anabolic one.^16^

This study further supports the hypothesis that distinct molecular patterns underlie HFrEF and HFpEF pathophysiology, extending previous knowledge to genes involved in somatotropic axis regulation, calcium handling, and adrenergic derangement in HF patients with stable clinical status.

## Conflict of interest

The authors declare that they have no conflict of interest.

## Funding

A.S. and L.B. were supported by a research grant provided by the Cardiopath Programme. A.S. received a research grant support from UniNA and Compagnia di San Paolo, as part of the programme STAR. A.M.M. was supported by an institutional grant from the Italian Healthcare Ministry (Ricerca Finalizzata for young researchers—project no. GR‐2016‐02364727).
